# Effect of Relative Humidity on Hydrogen Peroxide Production in Water Droplets – CORRIGENDUM

**DOI:** 10.1017/qrd.2021.11

**Published:** 2021-11-04

**Authors:** Maria T. Dulay, Carlos Alberto Huerta-Aguilar, Christian F. Chamberlayne, Richard N. Zare, Adriaan Davidse, Sinisa Vukovic

In the aforementioned article, an author’s ORCID ID was incorrect. The ORCID ID for Carlos Alberto Huerta-Aguilar was given as: 0000-0002-9987-0326 however it should be: 0000-0001-9274-5330. The authors apologise for this error.
